# Risk-Adapted Intraoperative Radiation Therapy (IORT) for Breast Cancer: A Novel Analysis

**DOI:** 10.1245/s10434-023-13897-3

**Published:** 2023-07-18

**Authors:** Melvin J. Silverstein, Brian Kim, Kevin Lin, Shane Lloyd, Lincoln Snyder, Sadia Khan, Katherine Kramme, Peter Chen

**Affiliations:** 1 Department of Surgery, Hoag Memorial Hospital Presbyterian, Newport Beach, CA USA; 2 Department of Surgery, Keck School of Medicine, University of Southern California, Los Angeles, CA USA; 3 Department of Radiation Oncology, Hoag Memorial Hospital Presbyterian, Newport Beach, CA USA

## Abstract

**Background:**

Randomized trials have shown that risk-adapted intraoperative radiation therapy (IORT) after breast-conserving surgery for low-risk breast cancer patients is a safe alternative to whole-breast radiation therapy (WBRT). The risk-adapted strategy allows additional WBRT for predefined high-risk pathologic characteristics discovered on final histopathology. The greater the percentage of patients receiving WBRT, the lower the recurrence rate. The risk-adapted strategy, although important and necessary, can make IORT appear better than it actually is.

**Methods:**

Risk-adapted IORT was used to treat 1600 breast cancers. They were analyzed by the intention-to-treat method and per protocol to better understand the contribution of IORT with and without additional whole-breast treatment. Any ipsilateral breast tumor event was considered a local recurrence.

**Results:**

During a median follow-up period of 63 months, local recurrence differed significantly between the patients who received local treatment and those who received whole-breast treatment. For 1393 patients the treatment was local treatment alone. These patients experienced 79 local recurrences and a 5-year local recurrence probability of 5.95 %. For 207 patients with high-risk final histopathology, additional whole-breast treatment was administered. They experienced two local recurrences and a 5-year local recurrence probability of 0.5 % (*p* = 0.0009).

**Conclusions:**

Whole-breast treatment works well at reducing local recurrence, and it is a totally acceptable and necessary addition to IORT as part of a risk-adapted program. However, the more whole-breast treatment that is given, the more it dilutes the original plan of simplifying local treatment and the less we understand exactly what IORT contributes to local control as a stand-alone treatment.

In recent years, there has been de-escalation of local treatment for breast cancer, with fewer mastectomies, less axillary surgery, hypofractionated courses of whole-breast radiation therapy, accelerated partial-breast irradiation (APBI), and finally, no breast irradiation at all for some of the most favorable patients.^1–7^ Intraoperative radiation therapy (IORT) has been one of those de-escalating tools. The goal of IORT is to give all the necessary radiation therapy in a single treatment at the time of lumpectomy, greatly simplifying local therapy.

When first introduced, about 20 years ago, a single IORT treatment replaced 30–35 standard radiation treatments. The benefits of reduced time, increased convenience, lower cost, less exposure to a hospital environment, and minimal side effects were significant. With the development of a range of hypofractionation techniques and more sophisticated forms of APBI, the gains have diminished somewhat.

Two prospective randomized trials, TARGIT-A^8^ and ELIOT,^9^ have reported acceptable long-term results for IORT as an alternative to whole-breast radiation therapy (WBRT) for selected low-risk breast cancer patients. Despite these data, IORT has not been widely adopted in the United States.

The decision to use IORT as part of the local treatment plan is based on preoperative imaging and needle biopsy features that meet preset institutional criteria together with patient desire to simplify the local treatment process and decrease potential side effects. Typically, IORT is given during the initial lumpectomy surgery without any knowledge of final histopathology. The patient is preoperatively counseled that if unexpected high-risk pathologic characteristics are discovered on final histopathology, additional treatment, such as whole-breast irradiation, re-excision, a combination of both, or mastectomy will be suggested, a strategy entitled risk-adapted IORT.

Patient data are generally analyzed by the intention-to-treat method.^10^ Patients remain in the treatment group to which they were assigned no matter what treatment they did or did not receive. There is generalized agreement that the intention-to-treat method is the best and fairest way to analyze prospective randomized trials. Crossovers, dropouts, and missing data are generally similar between groups. Randomization guarantees that factors such as age, tumor size, nuclear grade, and the like are well-balanced between groups. But intention-to-treat analysis has the potential to be misleading when studies analyze risk-adapted IORT that is not part of a two-armed randomized trial.

Risk-adapted IORT allows for the addition of whole-breast treatment based on unexpected high-risk final histopathology. The ELIOT^9,11–13^ and TARGIT-A^8,14,15^ trials and the current study used a risk-adapted approach. All three studies had somewhat different guidelines for adding additional treatment, and all three did so in differing proportions. Therein lies a problem when the results are interpreted and compared. Added whole-breast treatment works exceptionally well at lowering the rate of local events for low-risk patients, with reported recurrence rates of only 1 % at 10 years.^5,8,9^ The greater the proportion of IORT patients treated with whole-breast irradiation or mastectomy, the lower the local recurrence rate becomes.

Because final histopathology is unknown when IORT is initially given, the risk-adapted strategy makes perfect sense and is an important and necessary part of an IORT program. But the more additional whole-breast treatment that is given, the more it obscures the understanding of what IORT accomplishes as a stand-alone technique.

This report details the first 1600 patients who received risk-adapted IORT at our facility. We analyzed all the patients by the intention-to-treat method as well as by the subgroups that received additional whole-breast treatment or IORT only. Subgroup analysis can show what IORT accomplishes by itself when used without additional whole-breast treatment.

## Methods

The IORT treatment was administered to 1600 unique breast cancers in 1565 patients (35 bilateral) with a diagnosis of invasive ductal, invasive lobular, ductal carcinoma in situ (DCIS), or a combination of these at Hoag Memorial Hospital Presbyterian, Newport Beach, California. These patients were accrued to an institutional review board-approved tumor registry trial from June 2010 through December 2021. The trial was designed in 2010 and did not include collection of race or ethnicity. All the patients were women. The patient demographics are listed in Table 1.

**Table 1:** Characteristics of IORT Trial Cohort

Variable	N (%)
N	1600
*Tumor type*	
DCIS	320 20%)
Infiltrating Ductal	1140(71%)
Infiltrating Lobular	140 (9%)
Median follow-up (range)	63 months (4 Mo–12.5 Years)
Median follow-up ≥ 1 years	1553 (97%)
Median follow-up ≥ 5 years	884 (55%)
Median age (range)	66 years (40-95)
Median tumor span	16 mm
*Hormone receptor status*	
Estrogen receptor positive	1528 (96%)
Progesterone receptor positive	1368 (86%)
*Immediate versus delayed IORT *	
Immediate	1519 (95%)
Delayed	81 (5%)
*2017 ASTRO APBI categories*	
Suitable	729 (46%)
Cautionary	546 (34%)
Unsuitable	325 (20%)
*Biologic subtype (invasive only)*	
Luminal A	939/1280 (73%)
Luminal B (HER2 Neg)	277/1280 (22%)
Luminal B (HER2 Pos)	32/1280 (2.5%)
HER2 Pos	3/1280 (0.2%)
Basal	29/1280 (2.3%)

### Protocol Requirements

**Fig. 1:**
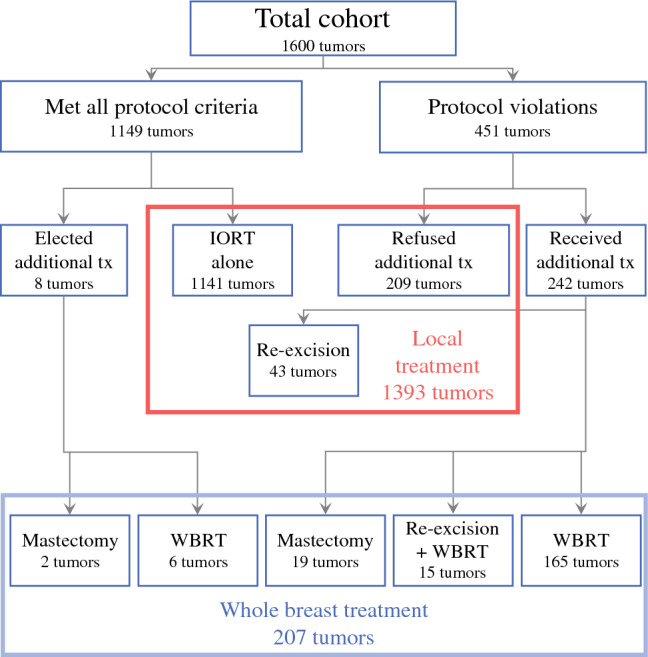
Flow chart outlining the different treatment groups. The group outlined in the RED box comprised 1393 tumors that received local treatment only. The group outlined in the BLUE box comprised 207 tumors that received whole-breast treatment. Tx, treatment; WBRT, whole-breast radiation therapy

For this study, the patients had to be 40 years old or older. Mammography, ultrasound, and MRI (unless medically contraindicated) were required for all the patients. Tumor extent had to be 30 mm or less in all imaging studies and by final histopathology. All invasive cancers required a negative sentinel lymph node by intraoperative frozen section. Final histopathology had to confirm a margin width of 2 mm or wider, negative axillary lymph nodes (N0 or Ni+ isolated tumor cells were acceptable), and no extensive lymphovascular invasion (LVI), defined as three or more foci and not multifocal/multicentric. Failure to meet any of these criteria was a protocol violation triggering a recommendation for additional risk-adapted treatment, which could be WBRT, re-excision, WBRT plus re-excision, or mastectomy. This resulted in five different treatment groups (Fig. 1). Additional treatment depended on the exact protocol violation and was determined by the treating team and patient.

### Procedure

After primary tumor excision, an IORT balloon and chest wall shield were placed, as previously described.^16^ Irradiation with 20 Gy (50 kV) x-ray was administered using the Xoft Axxent Electronic Brachytherapy System (Xoft, San Jose, CA, USA, a subsidiary of iCAD, Inc.).

### End Points

Recurrence was defined as any invasive or *in situ* event in any quadrant of the ipsilateral breast. Axillary and distance recurrences, breast cancer deaths, deaths from other causes, and side effects were recorded.^17^

### Statistical Analysis

Kaplan-Meier analyses were used to estimate local recurrence and survival probabilities. Log-rank testing was used to compare curves. A Cox proportional hazard model was used to examine recurrence hazard ratios for key characteristics. All analyses were performed using R Statistical Software (R Foundation for Statistical Computing, Vienna, Austria, v4.1.2; R Core Team 2021).

## Results

Between June 2010 and December 2021, 1600 tumors were treated. The median follow-up period was 63 months, with 1553 patients (97 %) followed more than 1 year and 884 patients (55 %) followed more than 5 years. The findings identified 1380 (80 %) of the tumors as invasive and 320 (20 %) of the tumors as DCIS. The median age of the patients was 66 years, and the median tumor size was 16 mm. According to American Society for Radiation Oncology (ASTRO) criteria, 46% of the tumors were suitable for APBI, 96% were estrogen receptor-positive, and 73% of the invasive tumors were luminal A.

For 72 patients, IORT was canceled in the operating room due to a positive sentinel lymph node (*n* = 59) or a skin-to-balloon distance less than 8 mm by ultrasound (*n* = 13). These patients are not included in this report. The average treatment time was 11 min. Of the 1600 patients, 1519 received IORT during the initial lumpectomy, and 81 received delayed IORT during a second surgery.

### Tumors that Met all the IORT Protocol Criteria

For 1149 (72%) of the tumors, all the Hoag protocol requirements were met, and 1141 of these tumors (99.5%) received IORT as their only local treatment. Although they met all IORT criteria, eight patients elected additional treatment. Six of the eight patients added WBRT, and two underwent conversion to mastectomy.

### Tumors that did not Meet all the IORT Protocol Criteria

The remaining 451 patients (28%) experienced a total of 573 protocol violations. The protocol violations and the additional treatment received are detailed in Table 2. All the patients who failed one or more criteria were advised to receive additional treatment. However, 209 patients (46%) refused any additional breast treatment. The remaining 242 patients (54%) failed one or more of the criteria and accepted additional treatment, namely, re-excision (*n* = 43), re-excision plus WBRT (*n* = 15), WBRT (*n* = 165), or mastectomy (*n* = 19). These five treatment groups can be simplified into two cohorts (Fig. 1; Table 2): those receiving local treatment (1393 patients receiving IORT only [*n* = 1350] or IORT plus re-excision [*n* = 43]), and those receiving whole-breast treatment (207 patients receiving IORT plus WBRT [*n* = 171], IORT plus re-excision plus WBRT [*n* = 15], or IORT followed by mastectomy [*n* = 21]). For 1350 patients, IORT alone was administered, including 1141 patients who met all IORT requirements and 209 patients who declined additional recommended treatment).

**Table 2:** Protocol violation and treatment

Protocol deviations	573 deviations in 451 patients
Margins < 2 mm	248
Tumor Extent > 30 mm	211
Positive lymph nodes	54
Extensive lymphovascular invasion	47
Multifocal/multicentric	13

Treatment following protocol deviations	451 Patients
Refused additional local treatment	209 (46%)
Accepted additional local treatment	242 (54%)
Re-excision alone	43
Re-excision plus WBRT	15
WBRT	165
Mastectomy	19

Treatment groups: all patients	1600 patients
Local treatment	1393 (87%)
IORT only	1350
IORT plus re-excision	43
Whole breast treatment	207 (13%)
IORT plus WBRT	171
IORT plus re-excision plus WBRT	15
IORT plus mastectomy	21

### Recurrence and Survival

There were 81 ipsilateral breast tumor recurrences: 61 invasive and 20 DCIS cases. Whereas 55 recurrences (68%) were in the same quadrant as the index cancer, 26 (32%) were in different quadrants.

The Kaplan-Meier probability of local recurrence for all 1600 patients at

**Fig. 2:**
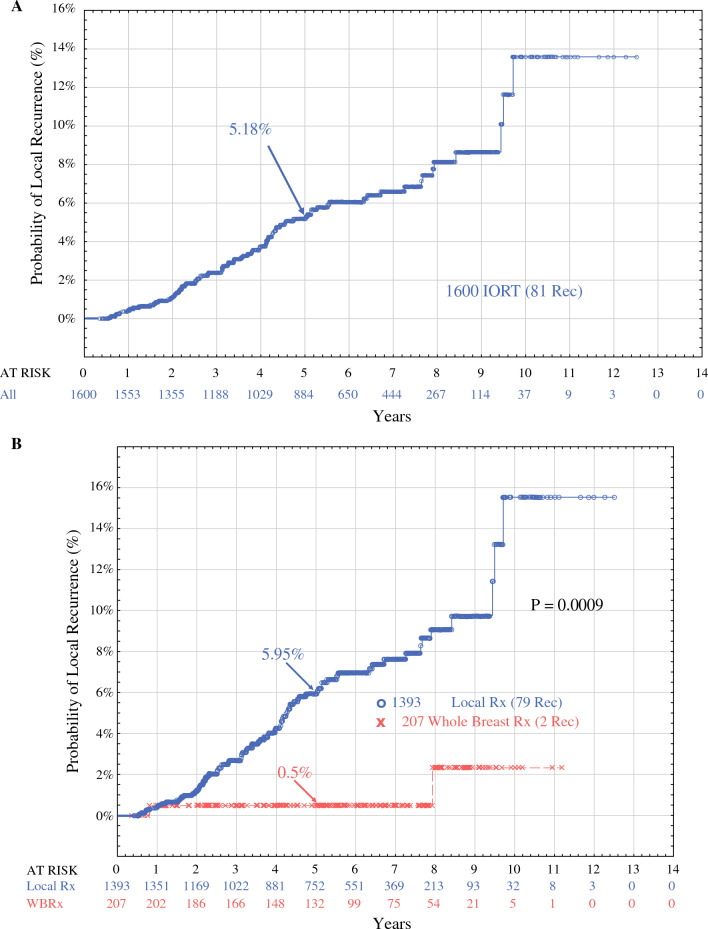
**A** All the treatments in 1600 IORT cases analyzed by the intention-to-treat method, with all 81 recurrences. The 5-year probability of local recurrence was 5.18 %. **B** The 1600 IORT cases were split into two groups: those who received local breast treatment (*n* = 1393) versus those who received whole-breast treatment (*n* = 207). The 1393 patients who received local breast treatment had a 5-year local recurrence probability of 5.95 % compared with 0.5 % for the 207 patients who received whole-breast treatment (*p* = 0.004), a difference of 5.45 %. The probability of local recurrence was only 0.5 % for those who received whole-breast treatment despite the fact that they were the most likely patients to experience recurrence because they had poor final histopathology requiring additional treatment. IORT, intraoperative radiation therapy

5 years was 5.18% (Fig. 2A). These patients were subdivided into two key groups: whole-breast treatment (*n* = 207) versus local treatment (*n* = 1393). The 5-year probability of local recurrence was 0.5% for whole-breast treatment and 5.95 % for local treatment (*p* = 0.0009; Fig. 2B). In a previous publication, whole-breast treatment was shown by multivariate analysis to be the single most important predictor of local recurrence.^18^

The 5-year probabilities of local recurrence, distant recurrence, and survival using a variety of subgroupings are reported in Table 3. The DCIS lesions recurred at a higher rate than invasive cancer, but the difference was not significant. The subgroup with the lowest rate of recurrence was the highly favorable one defined by Whelan et al.^4^ (age ≥55 years, T1 (tumors 20 mm or smaller), N0 (lymph node negative), grade 1 or 2 (low grade or intermediate grade) ≥1-mm margins, luminal A status, Ki67 ≤13.25%, treatment with adjuvant endocrine therapy). In our series, 324 such tumors treated with IORT had a 5-year probability of 1.83% for local recurrence. For 209 patients who failed one or more Hoag IORT criteria and declined additional local treatment, the 5-year probability of local recurrence was 9.01%.

**Table 3:** Kaplan-Meier 5-year probability of local or distant recurrence or survival for subgroups

Recurrence location and type	N	Local recurrences	5-year probability
All Local recurrences (DCIS + Inv) all quadrants	1600	81	5.18%
All Local recurrences (DCIS + Inv) same quadrant	1600	55	3.53%
Invasive local recurrences All quadrants	1600	61	3.79%
Invasive local recurrences same quadrant	1600	43	2.72%

All local recurrences by whole breast or partial breast treatment		Local recurrences	5-year probability
Whole breast treatment (received WBRT or mastectomy in addition to IORT)	207	2	0.49%
Received whole breast radiation therapy in addition to IORT but not Mastectomy	186	1	0.55%
Partial breast treatment (received IORT alone or IORT Plus Re-excision)	1393	79	5.95%

Pure DCIS patients		Local recurrences	5-year probability
DCIS tumors (All recurrences)	320	19	6.27%
DCIS tumors (Inv recurrences)	320	10	3.13%

Subgroups		Local recurrences	5-year probability
IORT only patients	1350	75	5.80%
Met IORT requirement received IORT Only	1141	58	5.17%
Whelan criteria	324	5	1.83%
ASTRO suitable	704	25	4.19%
Hoag criteria	1140	58	5.17%
Kunkler criteria	384	12	3.32
Luminal A	815	29	4.09%

Axillary and distant recurrences		Axillary or distant recurrences	5-year probability
Axillary recurrences	1600	8 (6 after 5 yrs)	0.13%
Distant recurrences	1600	7	0.43%

Survival	N	Deaths	5-year probability
Breast cancer specific survival	1600	3	99.8%
Overall survival	1600	68	96.8%

### Effect of Additional Treatment

**Fig. 3:**
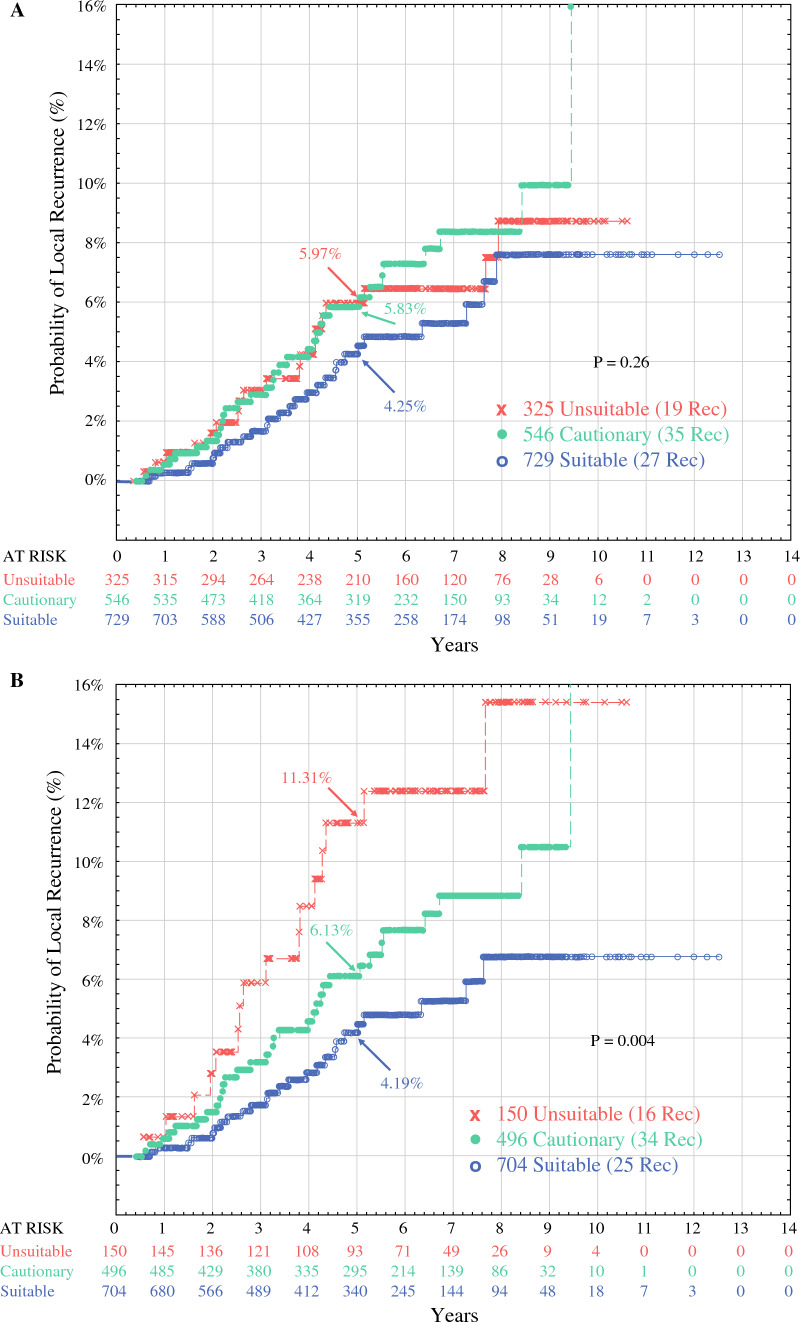
**A** All the treatments in 1600 IORT cases analyzed by the intention-to-treat using 2017 ASTRO criteria, with 81 recurrences. The value of the ASTRO categories was distorted by including 207 patients who received whole-breast treatment. Many whole-breast treatment cases were within the ASTRO-unsuitable group, making them similar to the cautionary group and blunting the value of the ASTRO categories. **B** The 1350 IORT-only cases analyzed per protocol using the 2017 ASTRO criteria. With removal of the patients who underwent whole-breast treatment, the curves separated, and the ASTRO categories became useful. IORT, intraoperative radiation therapy; ASTRO, American Society for Radiation Oncology

Additional whole-breast treatment dramatically decreases the probability of local recurrence, in some cases distorting the analysis. For example, when all 1600 patients were analyzed by the intention-to-treat method using the 2017 ASTRO criteria for APBI suitability,^19^ none of the curves differed significantly (Fig. 3A; *p* = 0.26). This suggests that the ASTRO categories were not predictive of patients who might benefit from IORT. However, many of the unsuitable category patients received whole-breast treatment, decreasing their probability of local recurrence and blunting the potential difference between curves. The analysis was repeated for 1350 patients treated with IORT only. The ASTRO category curves separated and became useful for choosing suitable patients for IORT (Fig. 3B; *p* = 0.004). For 150 ASTRO-unsuitable patients treated with IORT only, the 5-year probability of local recurrence was 11.31 %.

### Factors that May Play a Role in Local Recurrence

To better understand which factors play a role in local recurrence, we studied multiple variables using the cohort of 1350 patients who received IORT as their only local therapy. They experienced 75 local recurrences, with a 5-year recurrence probability of 5.80%. This cohort provided the purest way to analyze the impact of IORT alone. The factors analyzed included palpability, age, augmentation or not, invasive ductal versus invasive lobular carcinoma, invasive versus in situ carcinoma, specimen weight, adjuvant hormonal therapy, margin width, progesterone and estrogen receptors, nuclear grade, luminal A status, human epidermal growth factor receptor 2 (HER2) status, Ki67 ≤19 versus ≥20, and tumor span. The last six factors were independent predictors of local recurrence by univariate analysis. In the multivariate analysis, HER2 positivity and Ki67 of 20 % or higher remained significant (Table 4).

**Table 4:** Multivariate analysis of risk factors

Characteristic	Multivariate		
	HR	95% CI	*p*-value
HER2	3.09	1.32–7.28	0.010
Nuclear grade	1.18	0.72–1.95	0.511
Tumor span	1.02	0.99–1.04	0.180
Luminal A	1.20	0.56–2.59	0.638
Ki67	0.42	0.19–0.92	0.030
Estrogen receptor	0.72	0.32–1.65	0.442

## Discussion

This study tried to understand IORT in two contexts: (1) when used with a risk-adapted approach, analyzed by the intention-to-treat method, and (2) as a stand-alone treatment, analyzed per-protocol. The probability of local recurrence at 5-years for our entire cohort of 1600 tumors, when analyzed by the intention-to-treat method, was 5.18%. That number included the patients who met all criteria and received IORT only. It also included those who failed one or more criteria and accepted additional treatment as well as those who failed the criteria and did not accept additional treatment. In other words, it included all the patients regardless of treatment. As the proportions of each group changed, the result changed.

A risk-adapted approach is generally the analysis of choice for a randomized multi-arm trial because the percentage of dropouts, crossovers, and missing data usually is the same in all the groups. A single-arm study has a greater possibility of distortion because it has no second arm to balance treatment variations.

A more accurate assessment of individual patient risk might be developed if we combine the subgroup of patients who did not fail any criteria and received IORT as their only local treatment (*n* = 1141) with those who failed one or more criteria but accepted additional whole-breast treatment (*n* = 199). That would yield a subgroup of 1340 patients with a 5-year local recurrence probability of 4.41%. This is what patients can expect if they follow the risk-adapted guidelines. If a patient fails one or more criteria and does not accept additional breast treatment, the 5-year probability of local recurrence is more than twice as high at 9.01%. The ASTRO-unsuitable patients who refused additional whole-breast treatment had a recurrence probability of 11.31% at 5 years.

The study had several limitations. It was a prospective registry trial, not a randomized trial, so it had no standard treatment arm with which to compare outcomes. This trial was designed in 2009, and no data on race or ethnicity were collected, making it more difficult to translate our findings to the general population. Finally, the results for the patients who failed one or more IORT criteria were influenced by their willingness or unwillingness to accept additional treatment.

Whole-breast treatment works well at reducing local recurrence, and it is an important and necessary part of a risk-adapted program. However, the more we use additional whole-breast treatment, the more it defeats our original plan of simplifying local treatment and the less we understand exactly what IORT contributes. Analyzing risk-adapted strategies with intention-to-treat methodology can create a range of results, depending on what final histopathologic findings trigger additional treatment and what proportion they represent.

In this study, the contribution of whole-breast treatment was clear when we observed our patients who failed one or more IORT criteria and then received additional risk-adapted whole-breast treatment. These 207 patients made up our highest-risk cohort, with an expected 5-year recurrence rate of at least 9 %. But because they accepted whole-breast treatment, their 5-year probability of local recurrence was 0.5%, the lowest of any subgroup in our study.

Almost half of our patients (46%, 209/452) who failed one or more criteria did not accept additional local treatment when advised to do so. The most common reasons for failing IORT criteria were tumor larger than 30 mm or margins smaller than 2 mm. But TARGIT-A allowed no ink on tumor as adequate and a size up to 35 mm. When our patients reviewed the TARGIT-A protocol, they found it difficult to accept the addition of whole-breast treatment when they failed our criteria but were acceptable by TARGIT-A criteria. In addition, the patients who selected IORT often were patients who opposed whole-breast treatment from the beginning.

The TARGIT-A trial reported updated results in two separate papers: one for IORT given during the initial lumpectomy (immediate IORT)^8^ and one for IORT as a delayed secondary procedure.^20^ The IORT arm of the immediate TARGIT-A trial contained 1140 patients, with 65 receiving WBRT but no IORT, 38 receiving a mastectomy, and 241 receiving WBRT added to IORT. Of the 1140 patients, 344 (30.2%) received whole-breast treatment, substantially reducing the likelihood of local recurrence for the entire group. The TARGIT-A trial, using intention-to-treat methodology, reported the lowest 5-year probability of local recurrence in the literature (2.23%), but also the highest percentage of whole-breast treatment (30.2%).

The TARGIT-R trial,^21,22^ a North American trial using the same IORT delivery system as TARGIT-A reported a 5-year local recurrence probability of 6.6%, with 170 (25.1%) of 677 patients receiving WBRT. With a similar percentage of adjuvant WBRT and the same IORT delivery system, there is no explanation as to why the TARGIT-R recurrence rate is three times higher than with TARGIT-A. The ELIOT trial, which used electrons, reported a 4.2% recurrence probability at 5 years, almost twice as high as TARGIT-A, but only 5% of their patients received additional WBRT.^9^

The variability in local recurrence risk among published IORT trials is confusing to physicians and patients. Some of the problem stems from using intention-to-treat analysis in combination with varying percentages of risk-adapted treatment. Complicating the results further, histopathologic factors that trigger additional treatment varied between studies and even among different treatment centers in the same study.^8,14,20^

Recent literature has suggested the possibility of de-escalation using hypofractionation. The UK Fast-Forward^3^, the FlorenceTrial,^23^ and the Netherlands trial^24^ all have reported 5-years local recurrence rates ranging from 1.4 to 3.7% with 5 or 10 days of treatment. These results are similar to those of TARGIT A, but better than what we have experienced with IORT. However, without a randomized trial comparing IORT with any of these abbreviated courses of radiation, patients and physicians are left accepting the relative similarities in local recurrence and judging the toxicities of therapy as a possible deciding factor.

Further de-escalation can be achieved by selecting extremely favorable patients and skipping radiation therapy entirely. Kunkler et al.^5^ reported a randomized trial of 1326 women 65 years of age or older with ≤30-mm, node-negative, hormone receptor-positive tumors 30 mm or smaller in size and margins 1 mm or larger randomized to excision alone versus excision plus WBRT. All the patients received adjuvant endocrine therapy. At 10 years, the cumulative incidence of local recurrence was 9.5 % for the excision-alone group and 0.9 % for the radiotherapy group (*p* < 0.001). There was no difference in distant disease, overall survival, or breast cancer-specific survival. Whelan et al.^4^ have reported a similar but more restricted low-risk group (luminal A with Ki67 ≤13.25 %) treated with excision, no radiation therapy, and adjuvant endocrine therapy. They experienced a local recurrence probability of 2.3% at 5 years. Among our patients, 324 (20 %) met the Whelan et al.^4^ criteria and were treated with IORT alone. Their probability of local recurrence was 1.83% at 5 years. These results are encouraging as de-escalation moves forward. Although popular in Europe, IORT has not gained a strong foothold in the United States. No long-term data were available initially. This was not remedied until 2020, when acceptable 10-year data from TARGIT A and ELIOT^8,9^ were published.

A second major issue blocking widespread acceptance of IORT in the United States has been poor insurance reimbursement for both surgeons and radiation oncologists. That problem is not likely to be remedied.

A final issue making IORT difficult occurs when the radiation oncology team must leave the radiation oncology center and go to the operating room to administer the IORT. Although IORT takes only an average of 11 min to administer, the entire process often takes a total of 30 to 45 min of time away from the radiation oncology center. This problem can be compounded if there are operating room delays.

In the current era of de-escalating treatment strategies, physicians are faced with balancing the sacrifice of local control with the benefits of lesser side effects and better quality of life. In this de-escalation movement, IORT has tried to play a role but has found little acceptance in the United States. Although local control with IORT is inferior to that with WBRT, the benefits, including convenience, time saved, improved cosmesis, less exposure to hospital environments, and diminishment of late toxicities, cannot be ignored.^25^ With currently emerging long-term data showing acceptable local control results, IORT appears to be a possible solution to an otherwise all-or-nothing approach regarding adjuvant radiation in the management of breast cancer.
